# Knowledge, Implementation, and Perception of Enhanced Recovery After Surgery Amongst Surgeons in Pakistan: A Survey Analysis

**DOI:** 10.7759/cureus.46030

**Published:** 2023-09-26

**Authors:** Hamza Ahmad, Waqas Shehdio, Omaid Tanoli, Dan Deckelbaum, Tayyab Pasha

**Affiliations:** 1 Experimental Surgery, McGill University, Montreal, CAN; 2 Cardiac Surgery, Allama Iqbal Medical College, Lahore, PAK; 3 General Surgery, University of Toronto, Toronto, CAN

**Keywords:** enhanced recovery after surgery (eras), survey analysis, global surgery, eras protocols, enhanced recovery pathways

## Abstract

Introduction: An increasing shift towards non-communicable diseases and an existing high surgical burden of disease in low-middle-income countries (LMICs), such as Pakistan, has driven the need for implementing Enhanced Recovery After Surgery (ERAS), a safe and cost-effective surgical service aimed at improving patient recovery and reducing post-operative complications. Despite countless benefits, there are few ERAS programs throughout Pakistan and sparse literature on healthcare professionals’ views regarding ERAS. Without a deep understanding of healthcare professionals' perspectives on ERAS, underlying barriers and facilitators to a long-term ERAS implementation cannot be addressed and improved upon. Therefore, the purpose of this study is to better understand the knowledge, implementation, and perception of ERAS from the perspective of healthcare professionals across Pakistan.

Methods: Upon receiving ethical approval from the McGill University Health Center (MUHC), a previously validated questionnaire was modified and a 29-question survey was developed and disseminated to healthcare professionals practising in Pakistan. Quantitative data was analyzed using descriptive statistics and potential correlations that exist between the implementation of ERAS and the participants' gender, employment setting, and surgical specialty were investigated using the chi-squared analysis with a p-value of 0.05 as the cutoff.

Results: A total of 49 participants responded to this survey of whom 34 (69%) worked at a tertiary care teaching hospital whereas 15 (31%) worked at a private hospital. Surprisingly, 42 (85%) participants expressed being aware of the ERAS guidelines with only 30 (61%) either strongly agreeing or agreeing to successfully implementing ERAS into practice. The largest discrepancies in implementation were seen when discussing specific elements of the ERAS guidelines such as preoperative carbohydrate loading, practicing prolonged preoperative fasting, performing mechanical bowel preparation, performing active patient warming, and early postoperative removal of Foley’s catheter. Surgeons employed at a private institution were more likely to discuss postoperative pain management and control, less likely to utilize prolonged fasting, more likely to perform regular body temperature monitoring, more likely to practice providing chewing gum to patients postoperatively, and more likely to perform early removal of the Foley’s catheter.

Conclusion: An understanding of ERAS, the implementation of various elements, and a positive attitude toward its benefits definitely seem to be prevalent among healthcare professionals in Pakistan. However, key barriers and enablers specific to the underlying healthcare environment seem to be hindering the long-term successful implementation of ERAS across Pakistan. It is crucial for future studies to explore these barriers in further detail and involve the perspective of these key stakeholders to help enhance long-term ERAS adoption.

## Introduction

Enhanced Recovery after Surgery (ERAS) is a set of evidence-based perioperative guidelines that aim to improve patient care and recovery [1]. ERAS was first proposed by Danish professor of surgery, Henrik Kehlet, who questioned traditional perioperative care practices such as prolonged fasting, mobility limitations, mechanical bowel preparation, routine use of drains, and slow return to eating postoperatively [2]. Initial implementation of ERAS has shown that it not only lowers recovery time and postoperative complications as expected, but it is also a cost-effective method for reducing hospital and patient expenses [3]. Consequently, traditional protocols have been challenged by these scientifically-based guidelines and implemented globally in various surgical specialties, including colorectal, gastric, pancreatic, esophageal, bariatric, and non-gastrointestinal sub-specialties [1].

Globally,4.8 billion people do not have access to surgical care, with approximately 95% of this population being in low-middle-income countries (LMICs) such as Pakistan [4,5]. Hence, interest in implementing ERAS in Pakistan has risen, and several randomized controlled trials (RCTs) evaluating the potential benefits of ERAS have taken place, but without any long-term implementation being adopted across the country [6-8]. Potential barriers, such as resistance to change, standardization affecting personalized patient care, the buy-in of relevant stakeholders, information provision to patients, resources, palatability of nutritional drinks, aligning different ward cultures, patients going to non-ERAS departments, spreading the program within the hospital, differences in health issues, and utilizing a segmental approach have been identified in high-income countries (HICs) [9]. However, through rigorous research, reflection, and efforts to improve awareness regarding the long-term benefits of ERAS, challenges have been overcome, and several programs have successfully adopted ERAS in HICs [10-12].

A paucity of data regarding the knowledge, implementation, and most importantly, perception of healthcare professionals regarding ERAS in Pakistan could be a potential underlying barrier to long-term implementation. Therefore, the first step towards improving the standardization of ERAS in Pakistan is to determine the current familiarity and willingness of surgeons across Pakistan to apply the ERAS concepts in their individual practices. While barriers such as accessibility, availability, affordability, and acceptability of surgical care hinder improvements in LMICs, evidence suggests that interventions to improve surgical care in these settings can be cost-effective in the long term [13].

To that end, a survey was developed and disseminated to healthcare professionals across Pakistan to gain insight into their knowledge, implementation, and perception of ERAS. This context-specific information could provide insights into current practices and help identify key areas that could be used to establish and progress the necessary next steps toward improving the standardization of ERAS in Pakistan.

## Materials and methods

Participant recruitment

In January 2021, upon receiving ethical approval, a convenience sampling method was used to recruit participants who were available and willing to participate in the study by completing an online survey. A survey link using the secure REDCap tool was shared directly with the surgeons at various tertiary care institutions via email, obtained from various institutional administrations, and through several social media groups of surgeons across Pakistan. Informed consent, which included information concerning the purpose of the study and participant approval to use the data for the purpose of publication was obtained from the participants, and they had the choice to leave a question unanswered if the surgeon wished to do so. Participants were also assured regarding data anonymity and confidentiality. The inclusion criteria were being a surgeon practicing in Pakistan, regardless of gender or employment setting. 

Survey tool

A survey tool was developed to better understand the knowledge, implementation, and perception of healthcare professionals regarding ERAS. The 29-question survey was a modified questionnaire based on a previously utilized and validated survey for ERAS research (See Appendix) and pilot-tested amongst randomly selected faculty members in Pakistan [14]. The survey was broken down into three distinct sections for preoperative, intraoperative, and postoperative ERAS protocols. To ensure reliability, the survey utilized a Likert scale for the questions with the possibility of rating each item on a five-point scale from 0 (strongly disagree) to 5 (strongly agree). Only upon approval and finalization was the survey distributed to the participants.

Data analysis

After a one-month period of data collection and management using the REDCap system, the survey was closed. Descriptive and inferential statistics were generated using the data obtained from the survey. Individual surgeon demographics were dichotomized or categorized into multiple groups where appropriate. Chi-square tests of independence were performed to identify possible associations between demographic information and the implementation and knowledge of ERAS. Differences between groups were only considered significant when the p-value was < 0.05.

Ethics approval

Ethical approval was obtained from the McGill University Health Centre (MUHC) Research Institutional Board before study commencement (approval number: A10-B77-20A, 20-10-002).

## Results

A total of 49 responses were received from surgeons employed across the provinces of Punjab and Khyber Pakhtunkhwa. Out of the 49 respondents, male surgeons constituted a larger portion (n=31, 63%) compared to female surgeons (n=18, 37%). The majority of respondents (n=34, 69%) worked at tertiary care teaching hospitals, while the remaining worked at private hospitals (n=15, 31%). The city of Lahore accounted for the highest number of responses (n=38, 78%), followed by Peshawar (n=6, 12%) and Rawalpindi (n=5, 10%). General surgery was the predominant specialization among the respondents (n=22, 45%), with other specializations including cardiac surgery (n=10, 20%), orthopedic surgery (n=5, 10%), gynecology (n=4, 8%), otorhinolaryngology (n=3, 6%), urology (n=3, 6%), thoracic surgery (n=1, 2%), and pediatric surgery (n=1, 2%). The average work experience of the respondents was four years, ranging from a minimum of one year to a maximum of 15 years. Most respondents (n=33, 67%) had worked one to four years, while 14 (29%) had worked 5-10 years, and only two (4%) had worked for 11 years or more. Out of the 49 respondents, a majority (n=42, 86%) were aware of the ERAS guidelines. However, when it came to successfully implementing ERAS into practice, only 30 (61%) either strongly agreed or agreed to have adopted ERAS into practice.

Disparities were observed among individual practices regarding certain elements of the ERAS guidelines. For example, only 6 (12%) of the participants agreed or strongly agreed to provide carbohydrate loading preoperatively. A significant proportion 30 (62%) of the respondents agreed or strongly agreed to practice prolonged preoperative fasting. In terms of mechanical bowel preparation, 24 (48%) of participants agreed or strongly agreed, 16 (32%) were neutral, and only 6 (12%) disagreed with the practice. Only 19 (38%) of the participants agreed or strongly agreed to always performing active patient warming, and 27 (54%) agreed or strongly agreed to early postoperative removal of Foley's catheter to promote early mobilization. Detailed participant responses regarding individual elements of the ERAS guidelines are provided in Table 1.

**Table 1:** Responses to whether surgeons are always implementing individual ERAS elements ERAS: Enhanced Recovery After Surgery

ERAS Component	Strongly Agree	Agree	Neutral	Disagree	Strongly Disagree	Unanswered
Preoperative counseling is always performed	26 (53.06%)	18 (36.73%)	4 (8.16%)	1 (2.04%)	-	-
Discharge planning is always performed	15 (30.61%)	30 (61.22%)	-	3 (6.12%)	-	1 (2.04%)
Postoperative pain management and control are always discussed	14 (28.57%)	21 (42.85%)	2 (4.08%)	7 (14.28%)	2 (4.08%)	3 (6.12%)
Cessation of smoking 4 weeks prior to the surgery is always discussed	3 (6.12%)	25 (51.02%)	10 (20.40%)	5 (10.20%)	3 (6.12%)	3 (6.12%)
Underlying co-morbidities, including diabetes, hypertension, and coronary artery disease, are controlled and assessed prior to surgery	21 (42.85%)	10 (20.40%)	6 (12.24%)	7 (14.28%)	-	5 (10.2%)
Patients are always provided with carbohydrate-loading drinks preoperatively	-	6 (12.24%)	11 (22.44%)	20 (40.81%)	11 (22.44%)	1 (2.04%)
Preoperative fasting of solid and liquid food is commenced at midnight before surgery	17 (34.69%)	13 (26.53%)	1 (2.04%)	15 (30.61%)	2 (4.08%)	1 (2.04%)
Mechanical bowel preparation is Always performed preoperatively	6 (12.24%)	18 (36.73%)	16 (32.5%)	5 (10.20%)	1 (2.04%)	3 (6.12%)
Antibiotic prophylaxis is Always performed preoperatively	25 (51.02%)	18 (36.73%)	2 (4.08%)	1 (2.04%)	-	3 (6.12%)
Thromboprophylaxis is Always performed preoperatively	3 (6.12%)	21 (42.85%)	10 (20.40%)	12 (24.49%)	1 (2.04%)	2 (4.08%)
Regular body temperature monitoring is Always performed to avoid hypothermia	8 (16.32%)	28 (57.14%)	5 (10.20%)	7 (14.28%)	1 (2.04%)	1 (2.04%)
Active patient warming is Always performed	4 (8.16%)	15 (30.61%)	10 (20.40%)	18 (36.73%)	1 (2.04%)	1 (2.04%)
Multimodal opioid-avoiding analgesia is Always used for all patients along with regional anesthesia during surgery	5 (10.20%)	28 (57.14%)	10 (20.40%)	4 (8.16%)	-	2 (4.08%)
Customized and tailored analgesia is Always considered to enable the earliest possible transition to oral medications	4 (8.16%)	26 (53.06%)	13 (26.53%)	4 (8.16%)	-	2 (4.08%)
Chewing Gum is Always encouraged 3x/day for 5 minutes until the patient is tolerating food	3 (6.12%)	16 (32.65%)	14 (28.57%)	12 (24.49%)	4 (8.16%)	-
Given the patient is awake, alert, and able to swallow, early oral liquid and solid intakes are Always started within 4-6 hours postoperatively	8 (16.32%)	15 (30.61%)	7 (14.28%)	13 (26.53%)	4 (8.16%)	2 (4.08%)
Postoperative intrathecal opiates are used for pain management	1 (2.04%)	10 (20.40%)	9 (18.36%)	24 (48.98%)	2 (4.08%)	3 (6.12)
Foley's Catheter is Always removed in less than 48 hours postoperatively	12 (24.49%)	15 (30.61%)	11 (22.44%)	7 (14.28%)	2 (4.08%)	2 (4.08%)
Patients are recommended to start walking to prioritize early mobilization on post-operative Day 0	10 (20.40%)	20 (40.81%0	4 (8.16%)	11 (22.44%)	4 (8.16%)	-
Patients are recommended to start walking to prioritize early mobilization on post-operative Day 1	17 (34.69%)	26 (53.06%)	3 (6.12%)	3 (6.12%)	-	-

When examining the association between a surgeon's knowledge of ERAS and the successful implementation of ERAS using the chi-squared statistical analysis method, no significant correlations were found. However, when comparing individual ERAS elements with the surgeons' employment settings, with 15 (31%) surgeons appointed at private institutions and 34 (69%) appointed at public tertiary care teaching hospitals, several distinct associations emerged. Surgeons employed at private institutions were more likely than those at public tertiary care teaching hospitals to discuss postoperative pain management and control, less likely to utilize prolonged fasting, more likely to practice regular body temperature monitoring, more likely to provide chewing gum to patients postoperatively for bowel movement, and more likely to perform early removal of the Foley's catheter. Conversely, participants at tertiary care hospitals were more likely to perform preoperative active patient warming compared to participants at private institutions. Interestingly, participants employed at private institutions believed they were more successful at implementing ERAS compared to participants at tertiary care teaching hospitals. No significant associations were observed when comparing the gender, surgical specialty, or age of the surgeons to the implementation of various ERAS elements. These findings are further detailed in Table 2, which highlights the utilization of ERAS guidelines which were significantly different in private institutions when compared to the public tertiary care institutions. 

**Table 2:** Associations between ERAS elements and employment setting of surgeons ERAS: Enhanced Recovery After Surgery

ERAS Elements	Private Institution (%)	Tertiary Care Hospital (%)	Chi-Squared P-value
Discuss postoperative pain management and control	15 (100%)	20 (65%)	0.01
Utilize prolonged fasting	1 (6%)	29 (88%)	0.01
Practice regular body temperature monitoring	14 (93%)	22 (65%)	0.001
Provide chewing gum postoperatively for bowel movement	11 (73%)	8 (24%)	0.01
Perform early removal of Foley’s catheter	13 (87%)	14 (44%)	0.02
Believe in the successful implementation of ERAS	13 (87%)	17 (50%)	0.02

Finally, participants were asked to highlight key areas of focus that would help enhance the implementation of ERAS at their institution. These key areas of focus included enhanced management support, improved patient compliance, interdisciplinary collaborations, and surgeons’ knowledge and acceptance of ERAS. When questioned if ERAS should be implemented across Pakistan, only 10 (20%) participants agreed or strongly agreed whereas 20 (41%) were neutral, 17 (35%) disagreed or strongly disagreed, and two (4%) opted to not answer. Table 3 provides a summary of the responses.

**Table 3:** Participant responses for perceived barriers to the implementation of ERAS ERAS: Enhanced Recovery After Surgery

Barriers to the implementation of ERAS	Frequency
Enhanced management support	4 (8%)
Improved patient compliance	12 (24%)
Interdisciplinary Collaborations	16 (33%)
Surgeon’s knowledge and acceptance of ERAS	15 (31%)
Missing Response	2 (4%)

## Discussion

This study is the first that surveyed surgeons in Pakistan regarding their knowledge, implementation, and perception of ERAS guidelines. According to the results of this study, a vast majority of surgeons (86%) were aware of the ERAS guidelines and astonishingly 49% of the participants in the study agreed or strongly agreed to successfully implement ERAS into their practice. However, despite an impressive number of participants already implementing ERAS, several key areas of improvement were identified by the participants. One of the main areas of focus that could help improve the implementation of ERAS at their respective institutions was the surgeon’s knowledge and acceptance of ERAS. This finding is not surprising, as research conducted in HICs regarding healthcare professionals’ attitudes towards ERAS highlighted that the most common reason for resistance to ERAS tended to be the reluctance of healthcare professionals to accept ERAS and abandon traditionally taught protocols. [15] Similarly, participants also expressed that enhanced support from the management, improved patient compliance, and interdisciplinary collaborations could further aid the implementation of ERAS. It is important to note that these key areas of improvement identified by the participants encompass institutional and structural components, thus requiring the involvement of key stakeholders and policymakers to improve the awareness, acceptance, and implementation of ERAS across Pakistan.

Interestingly, specific elements of ERAS such as providing preoperative carbohydrate loading, practicing prolonged preoperative fasting, regularly performing mechanical bowel preparation, active preoperative patient warming, and early postoperative removal of Foley’s catheter tended to be the practices that were least adhered to. This finding not only highlighted that certain elements were harder to standardize but also shed light on the importance of addressing compliance to all the components of ERAS because minimum compliance of 80% to the ERAS protocol is associated with lower postoperative complications, shorter postoperative hospital stays, and improved patient satisfaction [16,17]. A policy that can be adapted to address healthcare professionals’ compliance with the different elements of ERAS is the use of an audit system, which can be imperative for achieving optimal patient outcomes [18].

When comparing the implementation of certain ERAS elements with the employment setting of the participants, interesting associations were observed, highlighting potential organizational barriers to the implementation of ERAS. Participants from private institutions were more successful at adhering to specific elements of the ERAS guidelines and believed to be more successful at implementing ERAS than participants at tertiary care teaching hospitals. Surprisingly, these findings are not in coherence with previous research which concluded that diagnostic accuracy and adherence to medical management standards were worse amongst the private sector care providers in Pakistan [19]. However, when taking into consideration that surgeons prefer to be employed at hospitals, which in turn focus on revenue generation through innovation and improved patient satisfaction, it is conceivable that there is an improved focus on implementing and standardizing ERAS protocols in the private sector.

Interestingly, enhancing management support does not seem to be a concern according to healthcare professionals when implementing ERAS. Instead, a concern is placed on patient compliance and a lack of interdisciplinary collaboration. Even though these barriers have also been identified in HICs, an emphasis on the lack of patient compliance has not been placed. This is an interesting phenomenon and one that requires further exploration through research. Without a patient buy-in into ERAS, interventions aimed at improving compliance might not be effective. Hence, it can be important before going forward to conceptualize and develop stronger educational programs to help patients understand the importance of ERAS and take an active role in its implementation.

Alongside highlighting the implementation of specific ERAS protocols, this study has provided a brief insight into the perceived barriers faced by healthcare professionals when implementing ERAS. However, a deeper understanding of these institutional, structural, and cultural barriers is still needed to better conceptualize and target policy that can help overcome such barriers. Qualitative studies in HICs aimed at better understanding the perspectives of healthcare professionals, executive leadership at the hospital, and patients helped develop a number of process-related implementation enablers, which ultimately led to the successful long-term implementation and standardization of ERAS across healthcare institutions [20]. Therefore, moving forward, studies should ideally focus on understanding the specific needs of healthcare professionals, executive leadership, management, and even patients when exploring a long-term implementation of ERAS in Pakistan.

Limitations

The inherent limitation of this study is that it was a survey study and is subject to biases such as nonresponse, selection (those to respond are more likely to have an interest and adopt), and information bias. Furthermore, a survey analysis is limited to the amount of information that can be gathered and only highlights some of the more superficial challenges faced by healthcare professionals. However, efforts were made to minimize the limitations of the study by approaching a large diverse participant pool with the inclusion of personnel from a myriad of working environments and developing a survey based on previously validated questionnaires for ERAS research. Nonetheless, a sample size of 49 and the majority of responses being mainly from the province of Punjab could impact the generalizability of the results obtained in this study. It is possible that healthcare professionals in other provinces and in different employment settings might have very different adaptations of ERAS. 

## Conclusions

The findings of this study suggest that a majority of surgeons are aware of the ERAS guidelines, and an impressive 49% of those surgeons are actually able to implement ERAS into their practice in Pakistan, which is just beginning to adopt ERAS. However, despite this initial start, several foci of improvement identified by the participants such as surgeons’ knowledge and acceptance of ERAS, interdisciplinary collaborations, improved patient education, and enhanced support from the management could further help implement ERAS in Pakistan. Currently, it can be inferred from the data of this study that these elements are hindering the implementation of ERAS and acting as barriers that can only be addressed if the administrative leadership, surgical leadership, and sociopolitical interests all align. Furthermore, implementing certain ERAS elements was more difficult than others; surgeons employed at private institutions were believed to be more successful in implementing ERAS compared to those in public tertiary care teaching hospitals. However, these differences were only limited to ERAS elements that are limited by financial abundance, whereas the key ERAS elements are being implemented at private and public institutions equally, highlighting the fact that resource availability is not a major barrier to the implementation of ERAS. Overall, it was clear that further research is still required to understand and address several institutional, structural, and cultural barriers to long-term ERAS implementation in Pakistan, especially because only a minority of surgeons agreed to have ERAS standardized across Pakistan. Understanding the reasoning and perspective behind this contradiction between implementation and willingness to standardize, and a lack of patient perspective towards ERAS can be potential topics for future studies. 

## Figures and Tables

**Figure 1:**
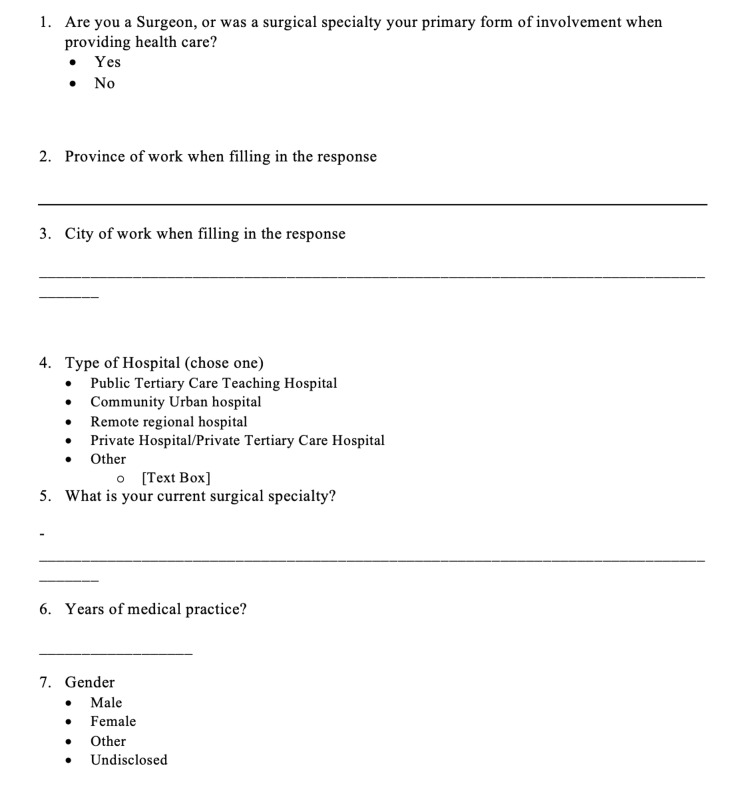
Questionnaire/Survey Page 1

**Figure 2:**
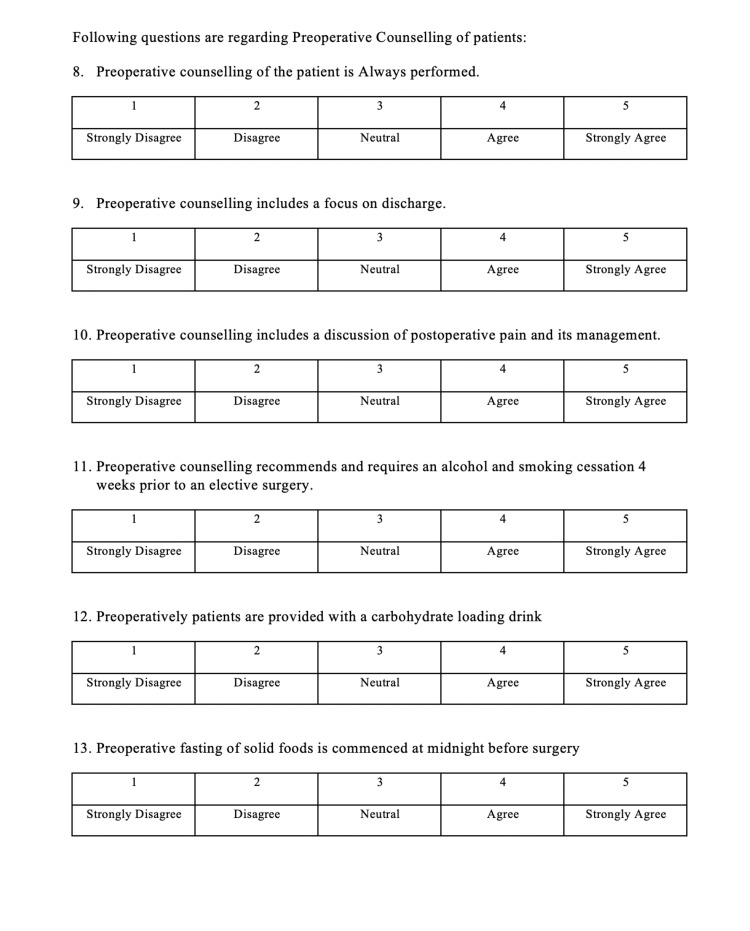
Questionnaire/Survey Page 2

**Figure 3:**
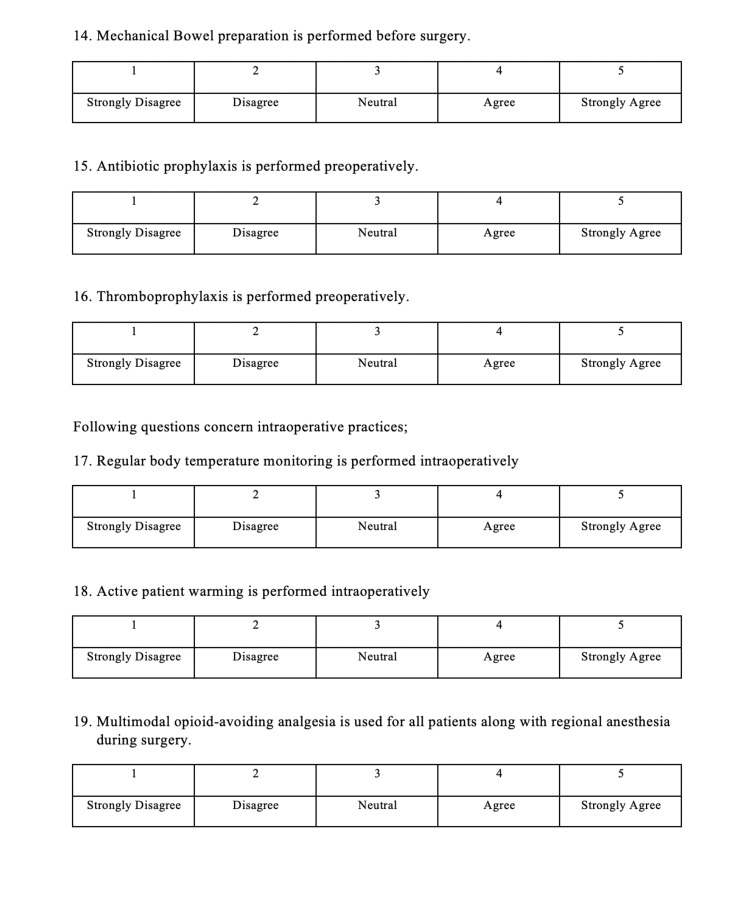
Questionnaire/Survey Page 3

**Figure 4:**
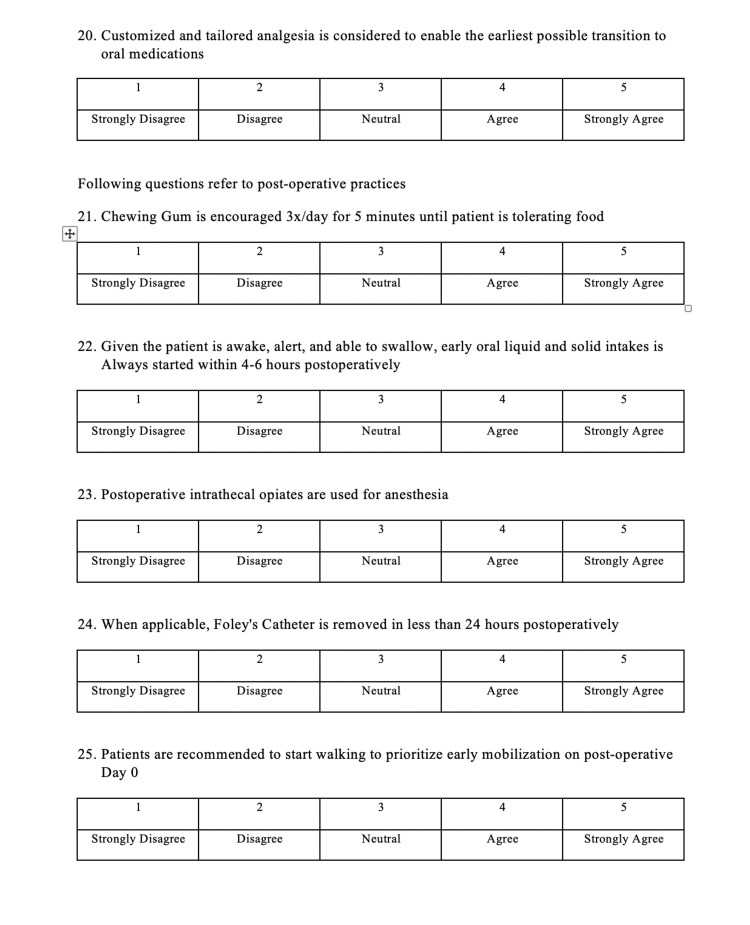
Questionnaire/Survey Page 4

**Figure 5:**
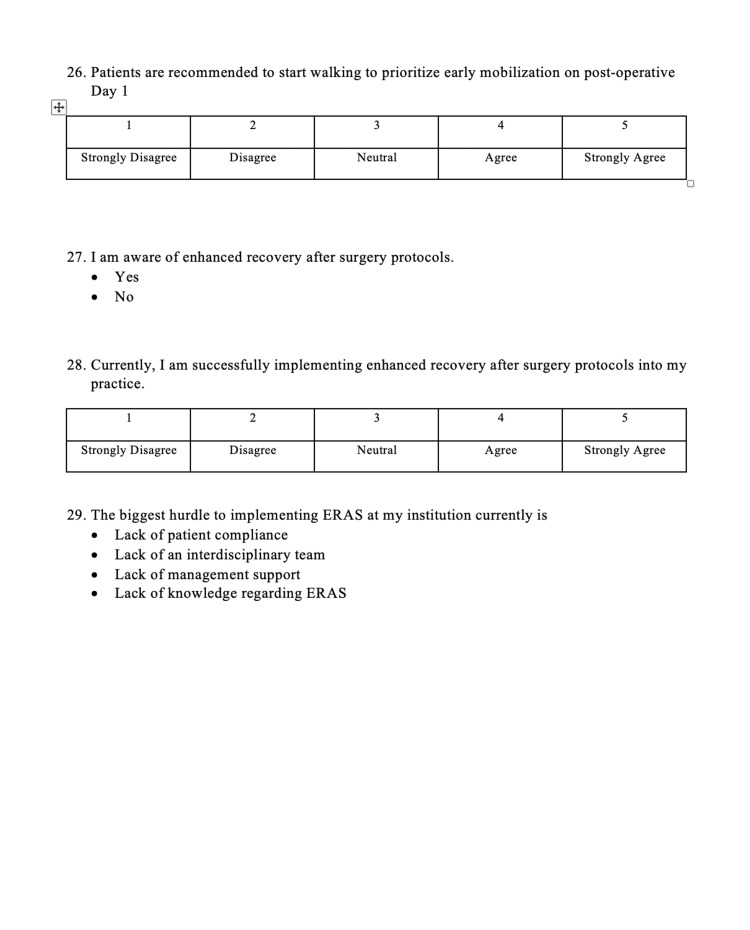
Questionnaire/Survey Page 5
